# Post translational modification regulation of transcription factors governing pancreatic β-cell identity and functional mass

**DOI:** 10.3389/fendo.2025.1562646

**Published:** 2025-03-11

**Authors:** Alicia Wong, Emilyn U. Alejandro

**Affiliations:** ^1^ Department of Genetics, Cell Biology, and Development, University of Minnesota Twin Cities, Minneapolis, MN, United States; ^2^ Department of Integrative Biology and Physiology, University of Minnesota Twin Cities, Minneapolis, MN, United States

**Keywords:** transcription factors, post-translational modification (PTM), pancreatic beta cells, pancreas development, beta cell proliferation, beta cell differentiation, diabetes, mature onset diabetes of the young (MODY)

## Abstract

Dysfunction of the insulin-secreting β-cells is a key hallmark of Type 2 diabetes (T2D). In the natural history of the progression of T2D, factors such as genetics, early life exposures, lifestyle, and obesity dictate an individual’s susceptibility risk to disease. Obesity is associated with insulin resistance and increased demand for insulin to maintain glucose homeostasis. Studies in both mouse and human islets have implicated the β-cell’s ability to compensate through proliferation and survival (increasing functional β-cell mass) as a tipping point toward the development of disease. A growing body of evidence suggests the reduction of β-cell mass in T2D is driven majorly by loss of β-cell identity, rather than by apoptosis alone. The development and maintenance of pancreatic β-cell identity, function, and adaptation to stress is governed, in part, by the spatiotemporal expression of transcription factors (TFs), whose activity is regulated by signal-dependent post-translational modifications (PTM). In this review, we examine the role of these TFs in the developing pancreas and in the mature β-cell. We discuss functional implications of post-translational modifications on these transcription factors’ activities and how an understanding of the pathways they regulate can inform therapies to promoteβ-cell regeneration, proliferation, and survival in diabetes.

## Introduction

Diabetes is a growing public health concern that affects an estimated 537 million individuals worldwide (1), a number that is projected to increase to 783 million by 2045 (2). 90% of diabetes cases are classified as Type 2 (T2D), which is characterized by insulin resistance (3), hyperglycemia, and loss of functional pancreatic β-cell mass (4). While conventionally thought mainly to be associated with ER stress mediated apoptosis, dedifferentiation, defined here as the loss of pancreatic β-cell identity as an insulin-producing cell, has increasingly been identified as another major driver of progressive β-cell failure in diabetes (5–9), a concept that has been extensively reviewed (10–17).

Developmentally, lineage determination, differentiation, and maturation, in the pancreas is controlled, in part, by activation of major transcription factors (TFs) and their interaction with gene regulatory networks (18). In the islets of Langerhans, Pdx1 (19) (Pancreatic Duodenal Homeobox 1) and Pax6 (20) (Paired Box 6), for example, both maintain pancreatic β-cell identity by suppressing genes that specify other islet cell types (19, 20). Functionally, as a nutrient-sensitive cell, it has been posited that signal-secretion coupling, where β-cells secrete insulin in response to flux in the nutrient milieu, relies on the collaboration between lineage-dependent TFs (ex. Pdx1) and signal-dependent TFs (ex. MafA) (21). Many of these TFs also undergo nutrient-dependent post-translational modifications (PTM), such as O-GlcNAcylation, which can alter their conformation, subcellular localization, stability, and activity (22).

In this review, we examine the role of PTM on major transcription factors governing pancreas and islet development and their role in maintaining the identity and function of pancreatic β-cells. We also examine their roles in β-cell identity loss in T2D, as well as how transcriptional activation of pathways during adaptive β-cell mass expansion events such as in obesity and pregnancy can be targeted in future β-cell regeneration therapies.

## Pancreas development

**Figure 1:**
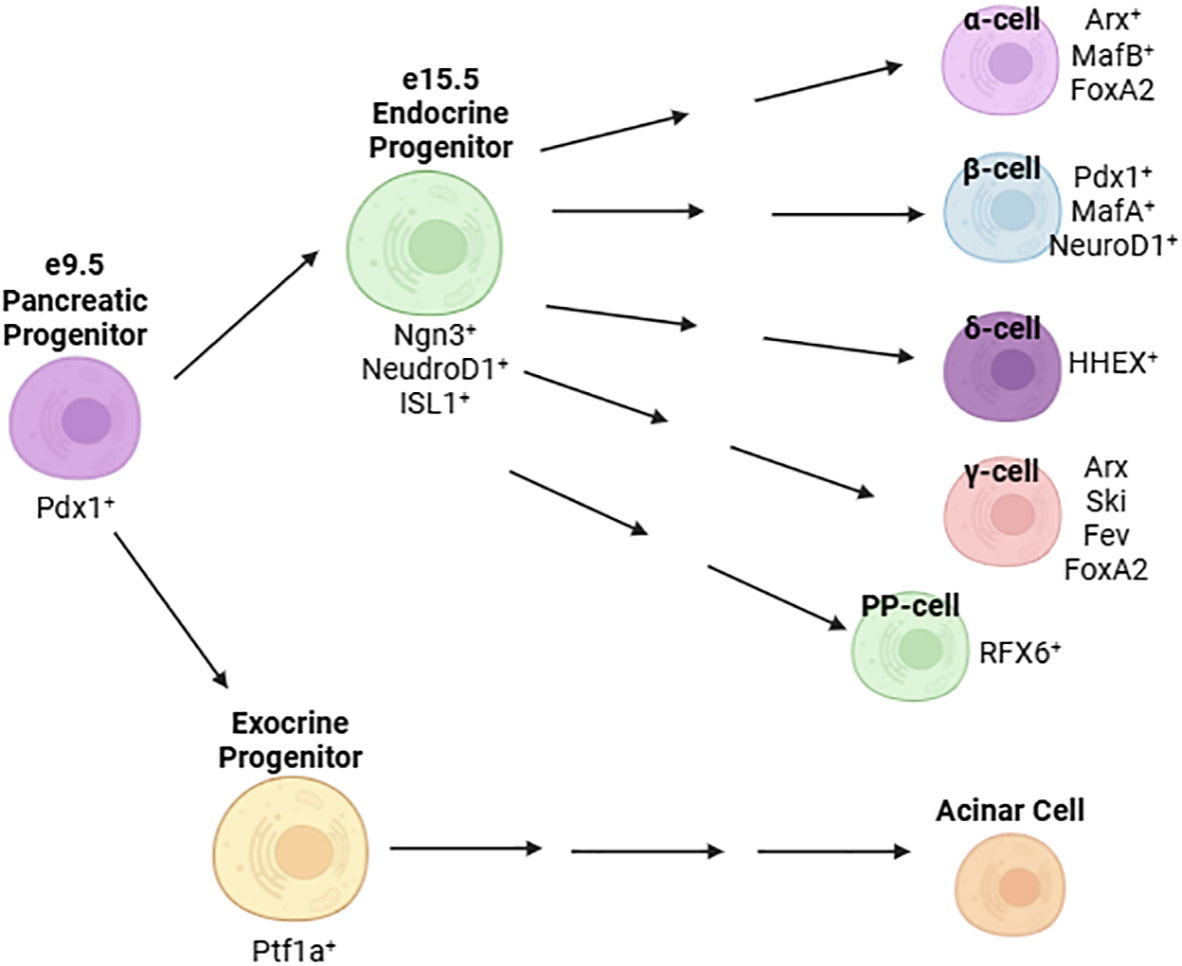
Major transcription factors defining the development and maturation of islet cells.

Pancreatic development is governed by a hierarchy of transcriptional activation. In the mouse, the pancreatic bud forms at embryonic day (e) 9.5 (23). At this stage, expression of master regulator Pdx1 is detectable on the foregut wall and specifies the pancreatic lineage (24, 25) (**Figure 1**). Deletion of Pdx1 at this point in development results in pancreatic agenesis in mice (26) due to uncoupling of the developing pancreatic epithelium from mesenchyme-derived morphogenesis signals (27). In humans, a homozygous point deletion that renders Pdx1 truncated and nonfunctional also causes pancreatic agenesis (28). By e15.5, the number of Ngn3^+^ endocrine progenitor cells peaks (29) and, together with expression of Isl1 (30), defines an endocrine islet cell fate (**Figure 1**) (25, 31, 32). Expression of NeuroD1 in these cells is required for differentiation into glucagon-secreting α-cells and insulin-producing β-cells (33). Later, the expression of Pdx1 and MafA are restricted to the mature β-cell (**Figure 1**).

## β-Cell “de-differentiation” in disease

Strong evidence from human and murine studies have tied the loss of β-cell mass in T2D to, in part, altered β-cell identity and function in response to a high glucose, high lipid environment rather than apoptosis alone (6, 34–37). Here, the term “de-differentiation” broadly refers to a β-cell that has lost its identity as an insulin-producing cell, rather than explicit reversion to a less mature stage of development.

Obesity is associated with insulin resistance and is a risk factor for the development of T2D (38–41). In both human obesity and high fat diet-fed rodent models, the increased demand for insulin is compensated for by an increase in β-cell proliferation and functional β-cell mass (42–44). In primary islets and in MIN6 cells, there exists a heterogeneous population of β-cells consisting of mostly “mature” cells expressing both the TF Pdx1 and a high level of insulin transcription (Pdx1^+^/Ins^high^) and a smaller population of Pdx1^+^/Ins^Low^ cells (45). In these Pdx1^+^/Ins^Low^ β-cells, genes characteristic of early β-cell development, namely MafB and Nkx2.2 are enriched (45). While these cells have a lower secretory capacity than the Pdx1^+^/Ins^high^ population, they have modestly improved proliferative capacity; a subset of them go on to become Pdx1^+^/Ins^high^ cells, suggesting Pdx1^+^/Ins^Low^ exist in a less mature developmental state more consistent with embryonic β-cell progenitors (45). Given these findings, it is possible that physiologically, this population of less mature but more proliferative β-cells is maintained to promote compensatory β-cell mass expansion in response to conditions such as obesity or pregnancy. Supporting this notion, chronic exposure to glucose *in vivo*, as demonstrated in rats, results in a compensatory expansion of β-cell mass to maintain euglycemia and also gives rise to a population of Pdx1^+^/Ins^Low^ cells (46). Given reduced transcriptional activity of Pdx1 in a diabetic milieu (47), it is feasible that this population of Pdx1^+^/Ins^Low^ cells is more susceptible to de-differentiation and cannot mount an effective compensatory response to a glucotoxic environment.

Other mechanisms have been proposed for β-cell de-differentiation in T2D. The forkhead TF, FoxO1, has been shown to be upregulated in islets in response to high fat diet feeding and orchestrates β-cell compensation in response to high-fat diet induced insulin resistance through expansion of β-cell mass (48). Knockdown of FoxO1 in murine β-cells results in defective compensation to physiological stressors such as successive pregnancies and aging (34). These mice experience reduced β-cell mass resulting from either the reversion of differentiated β-cells to a progenitor-like state or the trans-differentiation of β-cells to an α-like cell (34). More broadly, glucotoxicity and oxidative stress brought on by exposure to a diabetic milieu destabilizes the expression of TFs governing mature β-cell identity, including Pdx1 (49–51), MafA, and Nkx6.1 (51). As discussed earlier, these TFs have demonstrated roles in suppressing other islet cell programs.

There is evidence, however, that de-differentiation of β-cells is a potentially reversible process. In a mouse model of defective insulin secretion, lineage tracing studies demonstrate that β-cells revert to a Ngn3^+^/Insulin^-^ state under hyperglycemia but that these cells can re-differentiate back to a mature β-cell identity following normalization of blood glucose levels (37), suggesting de-differentiated cells retain significant plasticity. Wang and colleagues propose re-differentiation as a potential mechanism through which some T2D patients partially recover β-cell mass and function following long-term treatment with insulin (37). Further supporting the notion of islet-cell plasticity, severe loss of β-cells following ablation by diphtheria toxin is compensated, in part, by trans-differentiation of α-cells to augment β-cell mass (52). Additionally, there is evidence to suggest the conversion of α-cells to β-cells in mothers following pregnancy parturition in a murine model (53). Taken together, β-cell plasticity and differentiation capacity, as well as the TFs that govern this process, prove to be an attractive therapeutic target for preservation or reconstitution of functional β-cell mass in diabetes.

## Monogenic diabetes: a rationale to study transcription factors

Mature Onset Diabetes of the Young (MODY) describes a series of rare, heritable diabetic conditions that occur in young individuals and involves a dominant mutation of a single gene (54). Currently, 14 subtypes of MODY have been defined (54), half of which involve mutations in genes encoding transcription factors (**Table 1**). The causative link between loss of function mutations on TFs and the subsequent development of diabetes warrants further study on their regulatory role in pancreas/islet development and maintenance of glucose homeostasis in response to nutrient changes. Given that many of these TFs also undergo nutrient-dependent PTM (22), more efforts should be made in elucidating the molecular and biochemical effect of these PTM and the subsequence impact on β-cell survival and function.

**Table 1:** A summary of MODY subtypes involving mutations in transcription factors.

Subtype	Chromosome Location (human)	Gene
MODY 1	Chromosome 20 (55)	Hnf4a (56)
MODY 3	Chromosome 12 (57)	Hnf1a (58)
MODY 4	Chromosome 13 (59)	Pdx1 (60)
MODY 5	Chromosome 17 (61)	Hnf1b (62)
MODY 6	Chromosome 2 (63)	NeuroD1 (64)
MODY 7	Chromosome 2 (65)	KLF11 (66)
MODY 9	Chromosome 7 (67)	Pax4 (68)

## Modification of transcription factors governing β-cell identity and function in the mature islet

Post-translational modifications (PTM) are enzyme-catalyzed modifications onto the backbones or sidechains of translated proteins, often at specific amino acid residues (69). These PTM include phosphorylation (addition of phosphate group to Ser, Thr, Tyr), glycosylation (addition of sugar to Asn in N-linked, to Ser, Thr in O-linked), ubiquitylation (addition of ubiquitin to Lys), and acetylation (addition of acetyl group to Lys), among others (69). PTMs are a mechanism through which diversity of the proteome can be greatly increased apart from variations in amino acid sequence alone (69). These modifications can affect localization, function, and stability of target proteins (69), including many TFs in the pancreatic β-cell. Among them, Nkx2.2 (70), Nkx6.1 (71), and Pdx1 (19) are major players that govern islet development and play explicit roles in maintaining mature pancreatic β-cell identity through repression of genes conferring other islet cell fates. Other transcription factors, such as FoxM1 (72–75), play a role in maintaining or expanding functional β-cell mass. We examined these selected transcription factors and their post-translational modifications in detail. Other transcription factors involved in β-cell function and adaptation are summarized in **Table 2**.

**Table 2:** PTMs of transcription factors governing β-cell development, identity, and function.

TF	Function in Pancreas	PTM
Atf4	- Regulation of integrative stress response (152) - Maintenance of β-cell identity through ATOH8 (152) - Regulation of insulin production and glucose-stimulated ATP and cAMP production (153)	- Acetylation (154) - Methylation (155) - Phosphorylation (121, 156–158) - SUMOylation (159) - Ubiquitination (160, 161)
Cdx4	In zebrafish: - Determination of β-cell number (162) - Positioning of β-cells (162) - Positioning of foregut organs (162)	In mouse: - Phosphorylation (163)
Foxa2	- Binds to Pdx1 enhancer elements to regulate primordial pancreas expansion (164) - Vesicle docking and insulin secretion (165) - Terminal differentiation of α-cells (166) - Cooperative maintenance of β-cell fate (167)	- Acetylation (168, 169) - Phosphorylation (121, 170) - SUMOylation (171)
FoxM1	- Stimulates β-cell proliferation to maintain or expand β-cell mass (73–75, 130, 135) - Positively regulates insulin secretion (135)	- Acetylation (172) - Methylation (173) - Phosphorylation (121, 136–138) - SUMOylation (174, 175) - Ubiquitination (160)
FoxO1	- Promotes compensatory β-cell mass expansion in response to physiological stress (34)	- Acetylation (176, 177) - O-GlcNAcylation (178) - Phosphorylation (121, 176, 179) - Arginine Methylation (180)
Gfi1	- Development of pancreatic centroacinar cells (181)	- Methylation (182) - Phosphorylation (183) - Ubiquitination (160)
Glis3	- Cell lineage specification and β-cell development (184) - Regulation of insulin gene expression (184, 185) - Direct regulation of Slc2a2 and MafA expression (100)	- Phosphorylation (121, 186, 187)
Insm1	- Cooperation with binding partners NeuroD1 and FoxA2 to maintain β-cell maturity and function (167) - Regulation of β-cell specification in early postnatal development (188) - Repression of β to δ-cell transdifferentiation (189)	- Phosphorylation (121, 190)
Isl1	- Maintains postnatal β-cell function through direct regulation of Pdx1 and Slc2a2 transcription (191) - Controls both α-cell fate and β-cell maturation through epigenetic and transcriptional regulation of cell fate markers (192)	- Phosphorylation (121, 193)
MafA	- Activates insulin transcription through binding enhancer (194) and through synergistic interaction with NeuroD1 and Pdx1 (195) - Maintains mature β-cell phenotype (196)	- Phosphorylation (121, 197, 198) - SUMOylation (199, 200)
Mist1	- Regulates cellular proliferation and promotes terminal differentiation in exocrine pancreas development (201)	- Acetylation (202) - Phosphorylation (202, 203)
NeuroD1	- Required for islet morphogenesis (204) - Activates transcriptional network governing early differentiation of α and β-cells (33, 205) - Required for proliferation of perinatal α and β-cells (205) - Activates insulin gene transcription via interaction with p300, Pdx1, and E47 (206)	- Phosphorylation (207, 208)
Ngn3	- Specifies all the endocrine islet cell types during pancreas development (31) - Cooperates with Nkx2.2 to activate NeuroD1 (209)	- Methylation (210) - Phosphorylation (211, 212)
Nkx2.2	- Promotes terminal β-cell differentiation (113) - Organization of islet structure (115) - Regulation of insulin content and secretion (115) - Maintenance of β-cell identity through repression of Arx (70, 115, 116) - Cooperates with Ngn3 to facilitate activation of NeuroD1 (209)	- Phosphorylation (120, 121) - Ubiquitination (160)
Nkx6.1	- Establishment of β-cell fate (82) - Maintenance of β-cell identity and function through repression of other islet cell programs (71, 82)	- Methylation (213) - Phosphorylation (121)
Nrf2	- Mediates β-cell repair after high-fat induced oxidative stress (214) - Protects against oxidative stress proliferation of functional β-cell mass (215, 216)	- Acetylation (217) - Phosphorylation (218–220) - Ubiquitination (160, 221)
Pax4	- Essential for differentiation of β and δ cells (126, 222, 223) - Transcriptional suppressor of α-cell differentiation (224, 225) - Defines a subpopulation of proliferative β-cells (226)	- Phosphorylation (190)
Pax6	- Maintains β-cell identity by repressing alternative islet cell programs (20) - Transactivates insulin and glucagon promoters (227)	- Phosphorylation (228) - Ubiquitination (160) - SUMOylation (229)
Pdx1	- Defines a pool of pancreatic progenitors (26) - Suppresses α-cell genes to maintain β-cell identity (19) - Transactivates insulin promoter (24)	- Phosphorylation (49, 50, 98–101, 121) - O-GlcNAcylation (87–89) - SUMOylation (106)
Rfx3	- Regulates pancreatic endocrine cell differentiation (230) - Regulates β-cell maturation (222) - Mediates β-cell function through regulating β-cell glucokinase expression (222)	Phosphorylation (121, 186, 190, 231) - Ubiquitination (160)
Rfx6	- Maintains functional identity of mature β-cells through mediating glucose sensing, insulin secretion, and silencing disallowed genes (232)	- Phosphorylation (121, 190)
Ring1b	- Marks genes for repression in terminally-differentiated β-cells (233)	- Acetylation (234) - Phosphorylation (121, 235) - SUMOylation (159, 236) - Ubiquitination (160, 237, 238)
Sox9	- Maintains pancreatic progenitor cell pool (239) - Regulates alternative splicing of key genes in β-cell function (240)	- Acetylation (241, 242) - Methylation (243, 244) - Phosphorylation (245–247) - Ubiquitination (248)
Stat3	- When suppressed, promotes Pdx1-induced α to β-cell transdifferentiation following β-cell depletion (249)	- Acetylation (250–252) - Methylation (253–255) - Phosphorylation (121, 251, 256) - Ubiquitination (160, 161, 237, 248) - SUMOylation (257, 258)
Taf4	- Maintenance of functional β-cell identity (259)	- Methylation (213, 260) - O-GlcNAcylation (261) - Phosphorylation (121, 186, 262) - Ubiquitination (160, 161, 248)

All studies on function and PTM are conducted in human and/or rodent protein unless otherwise specified, but PTM events may not be specific to the β-cell. References to datasets from high-throughput proteomics studies are curated by PhosphoSitePlus (117).

### Pancreatic duodenal homeobox 1

Pdx1 is a master regulator of pancreas development, as well as β-cell function, identity, and survival. Originally coined Insulin Promoter Factor 1 (IPF1), Pdx1 was first identified as a novel insulin promoter binding protein expressed solely in the β-cell (76) and was found to transactivate both insulin (24) and somatostatin (77) gene transcription. Autoantibodies against Pdx1 have been detected in Type 1 diabetes (78), and mutations in Pdx1 are associated with increased Type 2 diabetes (T2D) risk (79). A dominant loss of function mutation in Pdx1 causes Mature Onset Diabetes of the Young (MODY) Type 4 (60). Later genetic studies confirmed that Pdx1 is indispensable for pancreas organogenesis (26, 27, 80). Genetic ablation of Pdx1 results in pancreatic agenesis by blocking outgrowth of the pancreatic bud and uncouples mesenchymal and epithelial pancreas development (26, 27, 80). β-cell-specific knockdown of Pdx1 disrupts glucose homeostasis and causes mature-onset diabetes in mice (81). Islets of mice with Pdx1-deficient β-cells have disrupted islet architecture and impaired Glut2 expression, accompanied by increased glucagon-expressing cells and insulin/glucagon co-expressing cells (81). Pdx1-deficient β-cells exhibit α-cell like ultrastructure, along with an α-cell-like electrophysiology and transcriptomic profile, including increased MafB and glucagon expression (19). In MIN6 cells, Pdx1 is found to bind upstream to the MafB coding region, and in Ins1 cells, depletion of MafB in the absence of Pdx1 is sufficient to prevent induction of glucagon (19), suggesting Pdx1 maintains β-cell identity by blocking an α-cell program through repression of MafB. Furthermore, the reduction of Nkx6.1 expression in Pdx1-deficient β-cells (81) suggests another mechanism by which Pdx1 maintains β-cell identity may be through the stabilization of Nkx6.1, which has been shown to repress α-cell factor, Arx (82).

Pdx1 is highly conserved among species. Its expression has been mapped to chromosome 13 (83) in humans and chromosome 5 in mice (84). Pdx1 is composed of two exons separated by a single intronic region, with no reported splice variants (85). Exon 1 encodes the amino terminus (85), which houses the transactivation domain (residues 13-73 in both mice and humans) (86), and Exon 2 encodes the carboxyl terminus (85), which includes the nuclear localization signal (residues 198-204 in mice, 197-203 in mice) (86). In MIN6 cells, Pdx1 has been shown to undergo the nutrient-sensitive O-GlcNAc modification (87–89), which has been shown to increase DNA binding affinity (87). In O-GlcNAcylation, the enzyme O-GlcNAc transferase (Ogt) catalyzes the addition of a single GlcNAc sugar molecule onto Ser and Thr residues of nuclear, cytoplasmic, and mitochondrial proteins (90, 91), and this modification is removed by O-GlcNAcase (Oga) (92). YinOYang, a server that generates neural network predictions for O-GlcNAc sites in protein sequences, has computationally predicted Pdx1 to be O-GlcNAc modified at T11, S273, and S274 (93). However, no studies to date have confirmed these findings. Much like the deletion of Pdx1, loss of Ogt in the β-cells results in progressive diabetes and reduced β-cell mass, accompanied by significant reductions in islet Pdx1 protein levels (94, 95). This was recapitulated in a mouse model of Ogt loss in the endocrine progenitors, where immunoreactivity to Pdx1 was reduced (96). Additionally, genetic ablation of Ogt in the pancreatic epithelial progenitors results in pancreatic aplasia (89), phenocopying pancreatic Pdx1 knockdown (26). Interestingly, in the absence of Ogt, overexpression of Pdx1 in the β-cells improves mitochondrial morphology and function (95), while normalization of Pdx1 levels in the pancreatic epithelium can partially restore pancreas weight and β-cell mass (97). Together, these studies provide indirect evidence for the positive regulatory role of O-GlcNAcylation on Pdx1. However, given the many O-GlcNAc modified proteins in the β-cell, further molecular studies are warranted to elucidate whether this regulation occurs because of a direct O-GlcNAcylation on Pdx1 or due to factors upstream.

In contrast, numerous studies have examined the effects of various phosphorylation sites on Pdx1 (49, 50, 98–101). Ser61 was found to be the principal site of phosphorylation by nanofluidic proteomic assays in both endogenous and overexpressed mouse Pdx1 (98). *In vitro*, phosphorylation at this site was found to be unchanged under both high and low glucose conditions, and despite existing in a phosphorylated state during embryonic development, expression of a phospho-dead mutant, Pdx1 S61A, had no adverse effect on pancreas development *in vivo (* 98), demonstrating the remarkable stability of this site under non-disease conditions. In contrast, under oxidative stress, which is associated with pathogenesis of T2D (102), increased phosphorylation of Pdx1 at several different residues targets it for degradation. Phosphorylation at S61 and/or S66 occurs in a glycogen synthase kinase 3 (GSK3)-dependent manner (49), while T11 is directly phosphorylated by Mammalian Sterile-20-like kinase (Mst1) (50), an amplifier of caspase-mediated apoptosis that is upregulated in a diabetic milieu (50, 103). In contrast, phosphorylation at T230 and S231 by CK2 increases Pdx1 transcriptional activity (104) through increasing Pdx1 stability (101). Pdx1 binds to E3-ubuiquitin ligase adaptor protein, SPOP, where it is targeted for ubiquitin-mediate proteasomal degradation (101). However, phosphorylation at T230 and S231 greatly decreases Pdx1 affinity for SPOP, allowing Pdx1 to maintain its function as a transcription factor (101). Taken together, these data implicate different phosphorylation events in the regulation of Pdx1 stability. In addition to protein levels, the localization Pdx1 in response to glucose is an important regulator of β-cell function. When the β-cell is exposed to high glucose, Pdx1 moves from the periphery of the nucleus to the nucleoplasm, where it can transactivate insulin transcription (105). *In vitro*, when S269 is phosphorylated by Homeodomain interacting protein kinase 2 (Hipk2), Pdx1 remains localized in the nuclear periphery (100), but further studies *in vivo* are necessary to characterize any effect on Pdx1 transactivation potential. Another mechanism governing Pdx1 localization is SUMOylation, the addition of small ubiquitin-like modifiers by Small Ubiquitin-related Modifier 1 (SUMO-1) (106). SUMOylation promotes both Pdx1 stability and nuclear localization; inhibition of SUMO-1 is associated with reduced transactivation of the insulin gene (106). Given the importance of Pdx1 in β-cell function and survival, targeted manipulation of Pdx1 PTM may inform therapies in maintaining β-cell function in diabetes.

### Nk2 homeobox 2

As a homeobox gene, Nkx2.2 plays a pivotal role in the development of the central nervous system (107–110). In mice, expression of Nkx2.2 is detectable in the developing forebrain beginning 9 days post-coitum (dpc) (111). Originally thought to be brain-specific, Rudnick and colleagues detected expression of Nkx2.2 and other homeobox genes in murine β-cell lines (112). This was later confirmed *in vivo* by Sussel and colleagues, who additionally found Nkx2.2 expression in both α and PP cells (113). Mice carrying a homozygous null mutation of Nkx2.2 lack β-cells and have reduced α and PP cells, resulting in severe hyperglycemia and neonatal mortality (113). Interestingly, there is a large population of partially-differentiated “β-like” cells that express Isl1 and Pdx1 but neither secrete insulin nor express other canonical β-cell markers such as Glut2 and Nkx6.1, suggesting Nkx2.2 is required for terminal differentiation of β-cells (113). Supporting this notion, when given a series of developmental transcription factors in a timed manner, ending with Nkx2.2, human fibroblasts can be differentiated into β-cells with functional glucose-stimulated insulin secretion both *in vitro* and when transplanted into immunodeficient mice (114). In the mature islet, Nkx2.2 plays a functional role in regulating both β-cell function and islet architecture. When Nkx2.2 is repressed in the β-cells in mice, there is a downregulation of MafA, a downstream target of Nkx2.2 and a key TF in β-cell maturation and glucose response (115). Furthermore, these mice are glucose intolerant, accompanied by impairments in insulin content and secretion, as well as disruptions in islet structure during islet assembly at e18.5 and persisting into adulthood (115). In addition to function, Nkx2.2 also plays a critical role in the maintenance of β-cell identity. RNA sequencing of islets from mice with Nkx2.2 deficient β-cells indicated repression of factors governing β-cell function, such as Glut2 (70, 115) and Nkx6.1 (70), and lineage tracing of these β-cells confirmed the co-expression of hormones associated with other islet cell types such as glucagon, somatostatin, and pancreatic polypeptide (70). Mechanistically, Papizan and colleagues demonstrate that Nkx2.2 directly binds the promoter of the canonical α-cell gene, Arx, where it is proposed to recruit its binding partner, co-repressor protein Grg3, to repress Arx expression (116). Furthermore, Nkx2.2/Grg3 also complexes with HDAC1 and Dnmt3a at the Arx promoter in β-cells, lending credence to the notion Nkx2.2 and Dnmt3a work together to repress Arx expression in the β-cell (116). Taken together, these data suggest that Nkx2.2 plays a pivotal role in maintaining β-cell identity as an insulin-secreting cell by repressing other pancreatic endocrine cell programs.

Most of what is known about post-translational modifications on Nkx2.2 are from large-scale proteomics datasets in human and mouse tissues. In both mice and humans, the DNA-binding domain of Nkx2.2 is located on amino acid positions 128-187 (86). While PTM in this region have not been explicitly studied in the pancreatic β-cell, Akimov et al. demonstrated in the Hep2 and Jurkat human cell lines that K137 is ubiquitinated (116, 117). The specific effect of ubiquitination at this residue on Nkx2.2 has not been examined. However, the homeostatic balance of ubiquitination and de-ubiquitination is generally considered important in protein turnover and quality control. Protein degradation is regulated through the ubiquitin-proteasome system (118), allowing clearance of dysfunctional or misfolded proteins (119). In addition, a proteomic study in human ischemic breast and ovarian cancer samples indicated phosphorylation sites on Y152 and S163, residues within the homeobox region (117, 120). The effect of these PTM on the Nkx2.2, particularly on DNA-binding activity, warrants further study, including whether these same sites are modified in the β-cell. In the islet-specific context, Sacco and colleagues conducted a phospho-proteomic study of MIN6 cells and, combining stimulated and unstimulated conditions, found amino acid residues S27, S63, S103, S107, S199 to be phosphorylated (117, 121). To understand the biological impact of these PTMs, site-directed mutagenesis and immunoprecipitation studies should be performed to confirm the proteomics results and to elucidate the effect of phosphorylation at these residues.

### NK6 homeobox 1

Nkx6.1, a member of the NK homeobox family, is involved in β-cell formation and differentiation (122) and plays a role in suppressing acinar cell fate during pancreatic development through antagonism of Ptf1a (123). In humans, Nkx6.1 expression is detectable in the neural tube at Carnegie Stage (CS) 12 (29-31 dpc) and in the dorsal bud of the developing pancreas at CS 13 (30-33 dpc) (124). In mice, at e10.5, Nkx6.1 expression can be detected across the entire developing pancreatic epithelium (122). However, starting at the secondary transition of pancreatic development at e12.5, expression becomes restricted, ultimately becoming detectable only in the insulin-positive cells by e15.5, corresponding to the peak in β-cell formation (122). Overexpression of Nkx6.1 in Ngn3^+^ endocrine progenitors results in a reduction of non-insulin producing islet cell types (α, δ, ϵ, PP) with no differences in overall proliferation rates, suggesting expression of Nkx6.1 favors the establishment of a β-cell fate (82). Conversely, Nkx6.1 loss in Ngn3^+^ cells upregulates the expression of α-cell-associated TF, Arx, in insulin^+^ cells in neonates, but not in e15.5 embryos (82), suggesting a role for Nkx6.1 in the maintenance, but not formation, of β-cells. This is further supported by data showing the requirement of Nkx6.1 in postnatal β-cell function. Conditional inactivation of Nkx6.1 in β-cells of adult mice is associated rapid-onset glucose intolerance, hyperglycemia, and reduced circulating insulin; accompanied by reductions in genes associated with insulin-secretion and β-cell proliferation (71). Both constitutive (82) and conditional (71) inactivation of Nkx6.1 cause β-cells to adopt a δ-cell-like identity (71, 82). This, along with the increased Arx expression in insulin^+^ cells during Nkx6.1 deficiency (82), lends credence to the notion that Nkx6.1 regulates β-cell identity, in part, through repression of other islet cell programs.

There is evidence to support that the regulatory role of Nkx6.1 is carried out in a spaciotemporal manner (71, 125). Full-body ablation of Nkx6.1 in mice results in deficiency of insulin-producing cells when examined after the secondary transition in β-cell development at e13 but not prior (122). In contrast, the deletion of NK homeobox family member, Nkx2.2, yields a lack of insulin-producing cells through the entirety of pancreas development (122). Furthermore, concomitant loss of Nkx6.1 in the absence of Nkx2.2 phenocopies Nkx2.2 loss alone, suggesting the regulatory role of Nkx6.1 occurs hierarchically downstream of Nkx2.2 (122). In Nkx6.1-deficient mice, reconstitution of Nkx6.1 in the Pdx1^+^ domain, but not in Ngn3^+^ domain, can rescue β-cell development, suggesting that in the specification of β-cell fate, Nkx6.1 expression is required prior to Ngn3^+^ endocrine progenitor cell commitment (56). This sets Nkx6.1 regulation of β-cell fate apart from Ngn3-dependent, lineage-specifying TFs, such as Pax4 (125, 126). Later studies would also indicate substantial redundancy between Nkx6.1 and its paralog, Nkx6.2, with equivalent biochemical activities governing β-cell specification. In the absence of Nkx6.1, ectopic overexpression of Nkx6.2 in Pdx1^+^ cells can rescue the formation and maturation of β-cells, including restoring the expression of key β-cell maturity markers MafA and Glut2, as well as endocrine differentiation co-factor, Myt1, which is normally reduced in the absence of Nkx6.1 (56, 127). These results suggest the differential regulatory role of Nkx6.1 and Nkx6.2 are primarily due to the time in which they become expressed during pancreas development.

Through a phospho-proteomics study in stimulated and unstimulated MIN6 cells, Sacco and colleagues revealed phosphorylation of several Serine residues on Nkx6.1: S228, S335, S353, S359, S364, and S365 all within the C terminus with the exception of S228, located upstream of the homeodomain within the repressor region (86, 117, 121). Interestingly, S335, S353, S359, S364, and S365 were found to be significantly regulated by drug or glucose stimulation. However, to date, no studies have specifically confirmed these phosphorylation events. The C terminus of Nkx6.1 houses a binding interference domain, which in the mouse, is located from residues 306-364, which greatly decreases the DNA binding affinity of its own homeodomain (128). Interestingly, all but one of the purported phosphorylation sites identified by proteomics falls within this binding interference domain, warranting further studies of Nkx6.1 phosphorylation on DNA-binding affinity of its target genes. Furthermore, the S228 residue is located within the repressor domain spanning residues 102-269 (86). Assessing repression efficiency of gene targets using phospho-mimetic or phospho-dead Nkx6.1 mutants at this residue would provide valuable insights on potential mechanisms governing Nkx6.1 function.

### Forkhead box protein M1

Transcription factor FoxM1 is associated with cellular proliferation and growth of various cancers (129), and in the β-cells, is required for maintenance-level (73) and compensatory proliferation in response to partial pancreatectomy (130). In the murine embryonic and neonatal pancreas, FoxM1 is expressed in the endocrine cells (73). While genetic ablation of FoxM1 in the embryonic pancreatic endoderm results in normal β-cell mass at birth, these mice experience a decline in β-cell mass over time due to defective β-cell replication, suggesting FoxM1 is indispensable for postnatal β-cell proliferation (73). Expansion of postnatal β-cell mass results from replication of existing β-cells (131), and turnover significantly declines with age (132–134). In murine islets, this is accompanied by reduced islet FoxM1 gene expression (135). Activating FoxM1 expression in aged islets induces β-cell mass expansion through increased proliferation (135). Additionally, in young mice, a lack of FoxM1 expression in the β-cells results in reduced glucose-stimulated insulin secretion and induction of FoxM1 expression improves glucose homeostasis (135), suggesting that in addition to regulating proliferation, FoxM1 also regulates β-cell function. Phosphorylation of FoxM1 has been extensively studied in non-β-cells. Generally, it is thought that phosphorylation of FoxM1 controls its stability, nuclear entry, relief of its N-terminal repressor domain, and recruitment of co-factors. For example, in fibroblasts, phosphorylation by Raf/MEK/MAPK signaling allows for the nuclear translocation of FoxM1 during the G2/M phase of the cell cycle (136). In various human cell lines, Pololike kinase 1 (Plk1) directly phosphorylates FoxM1 at C terminus residues S715 and S725 (137, 138), which are located within the disordered but highly conserved αβα region of the transactivation domain (TAD) (138). During G1 of the cell cycle, FoxM1 forms an auto-repressive homodimer, wherein the αβα region of the TAD interacts with the ββαβ motif on the N terminal repressive domain (NRD) (138). Conversely, phosphorylation at S715 and S725 disrupts these interactions, allowing the αβα motif to, instead, interact with the intrinsically disordered region (spanning residues 328 – 583 in humans) (138). This super-activated homodimer conformation, common in the S – G2/M phases of the cell cycle, promotes cellular division (138). In 293FT cells and mouse embryonic fibroblasts, FoxM1 is shown to be phosphorylated by ABL1 at Y575, which stabilizes FoxM1 half-life through inhibition of ubiquitin-proteasomal degradation (139).

In MIN6 cells, Sacco and colleagues’ phosphoproteomic approach defined phosphorylation sites at S329, S332, and S635, none of which were significantly modulated by secretion-stimulating drug treatments (121). While biochemical approaches are warranted to validate the phosphorylation of the aforementioned residues, given the role of FoxM1 in regulating cellular proliferation, it may be necessary to assess FoxM1 phosphorylation using β-cells treated with proliferation-promoting agents in order to elucidate regulatory sites.

## Transcription factor regulation of physiological β-cell mass expansion

Expansion of functional β-cell mass through proliferation or regeneration has been a long-standing goal in the treatment of diabetes. Physiologically, β-cell mass has been known to expand in response to increased metabolic demand, such as in pregnancy (140–143) and obesity (4, 42, 144, 145). An understanding of the mechanisms governing this adaptive process can inform novel targets for future β-cell therapies. In the early stages of obesity, there is evidence for increased β-cell hyperplasia to compensate for increased insulin demand in non-diabetic individuals (4, 42, 144, 145). Using a non-diabetic mouse model of obesity, Leptin^ob/ob^, Davis and colleagues identified upregulation of islet transcription factor, FoxM1, accompanied by higher circulating insulin levels and lower plasma glucose (74). Expression of FoxM1 can trigger proliferation in both murine and human donor islets through activation of the cell cycle, and like in mice, its expression is upregulated in islets of obese, non-diabetic human individuals (74). This, along with the lack of FoxM1 upregulation in diabetic Leptin^ob/ob^ islets, provides evidence that FoxM1-mediated β-cell proliferation is necessary for the compensatory regulation of glucose homeostasis under obesogenic stress (74).

Normal pregnancy is associated with maternal insulin resistance, necessitating greater insulin demand, and consequently, the upregulation of β-cell mass (140, 146, 147). This has been demonstrated extensively in rodent models (140–143). During pregnancy, β-cell FoxM1 expression is upregulated and has been shown to be a downstream effector of placental lactogen, though the exact mechanism is not entirely clear (75, 141). Lack of FoxM1 expression in the pancreas results in gestational diabetes, associated with inadequate compensatory β-cell proliferation (75). In mice, epidermal growth factor receptor (EGFR) signaling has been shown to orchestrate the pro-survival and proliferative effects of placental lactogen on β-cells during pregnancy (148), and serotonin has been shown molecularly and transcriptomically to act downstream of placental lactogen signaling to promote β-cell proliferation (149, 150). Furthermore, treatment of human immortalized β-cells with serum from pregnant human donors significantly increased rates of proliferation, indirectly supporting the role of pregnancy-specific circulating factors in the upregulation of β-cell expansion in humans (151). Unsurprisingly, a comparison of the islet transcriptome in pregnant and non-pregnant mice revealed an upregulation of genes regulating cell growth, proliferation, and apoptosis, as well as genes governing insulin secretion and secretory granule biosynthesis (150). Taken together, these data support highly orchestrated genetic and hormonal regulations of β-cell mass expansion during pregnancy. Continued research to isolate and modulate key factors during this process could inform therapies to promote β-cell proliferation while simultaneously preventing cell death in the treatment of diabetes.

## Concluding remarks

In adults, new β-cells are formed by replication of existing β-cells rather than by differentiation of stem cells (263). However, the rate of proliferation to maintain basal β-cell mass is low (72). For this reason, targeting the health of existing β-cells or generating new functional β-cells *in vitro* have become two major focuses in the development of potential therapies for diabetes. TFs play an indispensable role in regulating pancreas and islet development and can serve as potential target in rescuing, preserving, or re-generating β-cells. Enhancing the expression of certain transcription factors via the addition of pharmaceutical compounds can have a protective effect on β-cells. For example, the flavonoid compound, tectorigenin, has been shown to enhance Pdx1 expression and protect β-cell viability under glucolipotoxic conditions (264). On the other hand, the successful generation of β-cells through differentiation of stem cells via the controlled addition of TFs has caused much excitement (114, 265, 266). Recently, islets derived from chemically induced pluripotent stem cells were successfully transplanted and engrafted into a human patient (267).

In the β-cell, many signal-dependent TFs cooperate with other proteins in response to stimuli, such as nutrient flux, to regulate adaptation responses (21). Many of these TFs also undergo nutrient-sensitive PTMs, which may influence their localization, stability, and function (22). However, while there are many loss-of-function studies examining the role of these TFs in the cell, few look at the stimulus-sensitive molecular mechanisms regulating the TFs themselves, such as PTM or epigenetics (not reviewed here). A deeper understanding of these elements can aid in developing more refined diabetes treatments targeting the pancreatic β-cell.

## Glossary

Atf4: Activating Transcription Factor 4
ATOH8: Atonal Basic Helix-Loop-Helix Transcription Factor 8
Cdx4: Caudal Type Homeobox 4
ER: Endoplasmic Reticulum
FoxA2: Forkhead Box Protein A2
FoxM1: Forkhead Box Protein M1
FoxO1: Forehead Box Protein O1
Gfi1: Growth Factor Independent 1 Transcriptional Repressor
Glis3: GLIS Family Zinc Finger 3
GSK3: Glycogen Synthase Kinase 3
Insm1: Insulinoma-Associated Protein 1
Isl1: Insulin Enhancer Protein Islet 1
MafA: MAF BZIP Transcriptional Factor A
Mist1: Basic Helix-Loop-Helix Family Member A15
MODY: Mature Onset Diabetes of the Young
NeuroD1: Neuronal Differentiation 1
Ngn3: Neurogenin-3
Nkx2.2: NK2 Homeobox 2
Nkx6.1: NK6 Homeobox 1
Nrf2: NFE2 Like BZIP Transcription Factor
Pax4: Paired Box 4
Pax6: Paired Box 6
Pdx1: Pancreatic Duodenal Homeobox 1
Plk1: Pololike Kinase 1
Rfx3: Regulatory Factor X3
Rfx6: Regulatory Factor X6
Ring1b: Ring Finger Protein 2
PTM: Posttranslational Modification
Sox9: SRY-Box Transcription Factor 9
Stat3: Signal Transducer and Activator of Transcription 3
T2D: Type 2 diabetes
Taf4: TATA-Box Binding Protein Associated Factor 4
TF: Transcription Factor
